# Cross-Domain Communication Method Based on Load Balancing for SDNs

**DOI:** 10.3390/s25041080

**Published:** 2025-02-11

**Authors:** Xiaomao Wang, Yi Zhou, Feng Dan, Xian Yang

**Affiliations:** 1 Engineering Research Center for Metallurgical Automation and Measurement Technology of Ministry of Education, Wuhan University of Science and Technology, Wuhan 430081, China; zhouyi83@wust.edu.cn (Y.Z.); danfeng@wust.edu.cn (F.D.); 2 Alliance Business School, University of Manchester, Manchester M13 9PL, UK; xian.yang@manchester.ac.uk

**Keywords:** software defined networking (SDN), path selection, load balancing, cross-domain

## Abstract

In multi-end-to-end path request planning, the control plane may not be able to meet all path request requirements under limited bandwidth resources. Moreover, suboptimal path planning can lead to localized network congestion, which in turn causes an overall imbalance in network load. Therefore, the multi-domain control plane needs to consider more network resource states during the path selection, such as link weights, load saturation, and resource occupancy rates, in order to select the optimal paths to maximize the satisfaction of data plane requirements while maintaining network load balance. To address such issues, we first derive a cross-domain communication load balancing objective function based on network modeling. Through collaborative processing among multi-domain controllers, the coordinated planning of cross-domain paths and the collaborative installation of flow tables are achieved. Then, we transform the cross-domain path planning problem into a clique-finding problem under a set of backup paths. Finally, we provide a heuristic approximate solution method for this problem. In terms of cross-domain communication, we adopt a collaborative approach among multiple controllers to achieve coordinated planning of cross-domain paths and collaborative installation of flow tables. Simulation results show that our proposed scheme outperforms the traditional method in terms of path allocation success rate, network load balancing degree, and data transmission delay, especially in cross-domain communication under high-density path requests in SDN networks.

## 1. Introduction

Software defined networking (SDN) belongs to centralized control. During the path allocation process, the control plane needs to consider the selection and optimization of end-to-end paths across the entire network, which puts new demands on the precision of the controller’s path selection and its global consideration. The traditional shortest path first (SPF) algorithm can no longer meet these requirements. Firstly, for any flow request, the controller cannot guarantee that there will always be a suitable path to meet the transmission requirements under the current state. Secondly, under bandwidth limitations, the end-to-end path selected based on the whole network topology view is not necessarily optimal. Lastly, even when there is sufficient remaining bandwidth, the selected path may lead to network load imbalance and network congestion.

In the case of missing flow table entries, the controller may receive multiple end-to-end path requests within an extremely short time. Due to limited network resources, the actual number of paths that can be allocated may not be able to meet all end-to-end path requests. In traditional BGP networks, routers do not consider the overall network load balance when finding routes, whereas in SDN, this new type of network, the overall network load balance is one of the important indicators to consider. When the existing resources are insufficient to meet all path requests, how the controller plans the path allocation scheme to maximize user satisfaction without causing network load imbalance is particularly important. Therefore, each domain controller needs to consider end-to-end flow requests from a global perspective. For multiple end-to-end path requests under limited resources, the controller should be able to select as many available paths as possible while ensuring that the network load is relatively optimal.

In the aspect of SDN path selection, Zongyu et al. [1] proposed an integrated optimization approach that combines adaptive load-balancing and heuristic path selection and used a deep learning model to predict the payload data and the convolutional neural network (CNN) model to predict the noise data. Based on the network architecture of the compute-first network (CFN), Bo et al. [2] analyzed the selection and decision-making process of traffic forwarding paths from edge networks to computing centers in CFN according to the actual enterprise business deployment requirements and combined the technologies of SD-WAN, SRv6, etc., to realize an end-to-end forwarding path selection model in compute-prioritized NICs and put forward an end-to-end forwarding path quality detection method. Li et al. [3] implemented dynamic selection of a single path and network load balancing by calculating the number of path hops, packet forwarding counts, byte forwarding counts, and port forwarding rates at both ends of the link. Celenlioglu et al. [4] reduced the controller’s time in routing by setting up multiple paths in advance to support multi-path routing in SDN. Ghaffarinejad et al. [5] combined the concept of load balancing at the application layer in traditional networks with the network layer through the use of SDN, thereby eliminating the traditional central control center. In [6], Giorgetti et al. introduced OpenFlow concepts to address the optimal path selection problem in optical networks, providing a solution approach for the GMPL problem. [7] proposed a distributed management program for dynamic path control. Alagitz et al. [8] proposed a demand-driven DynPaC framework for dynamic management of path allocation tasks. To address the issue of long propagation delays between controllers and switches, Lin et al. [9] proposed a path label routing method based on OpenFlow. In [10], Sheu et al. improved the traditional SPF and proposed a path selection algorithm, applying it to SDN networks. In [11], Huang et al. proposed a new multicast tree-based routing algorithm, proving that the research problem is NP-Hard and providing parameters that are not approximable within *k*. Ankit et al. [12] proposed the ELBA algorithm for multimedia data, utilizing SDN’s dynamic rerouting capabilities to stream different layers of SVC-encoded video on potentially different suitable paths. Lu et al. [13] proposed a new inter-domain multi-path flow transfer mechanism based on SDN and multi-domain collaboration, designing an information exchange method to maintain network status (e.g., inter-domain topology updates, link loads) through a multi-layer iterative detection method based on BGP notifications and inter-domain collaboration analysis. Rocha et al. [14] proposed a path computation model, establishing the integration between data path deployments between servers based on the model’s principles. Lamali et al. [15] proposed a corresponding path selection algorithm by considering the bandwidth constraints of each link and ensuring Quality of Service (QoS). In [16], Trivisonno et al. proposed a method to maximize the number of link requests, which can select the maximum number of links that meet the conditions from multiple specific link requests based on broadband limit conditions. Llopis et al. [17] improved the processing timeliness of key businesses by identifying paths with minimal delay in real-time and routing important data services to those paths.

In terms of routing load balancing, Naga Katta et al. [18] proposed a method to dynamically adjust loads by tracking congestion on the best paths to destinations between adjacent switches within the data plane, thereby achieving better network load balancing by reducing the generality of switches. Salvestrini et al. [19] achieved dynamic load balancing through distributed controllers and inter-platform signaling in SDN. In [20], the authors proposed a method to reduce the load on HTTP servers using OpenFlow, mainly by customizing flow routing to reduce network and server response times. In [21], Mi et al. proposed local routing and management of node status based on controller-based mobile agents, preventing excessive traffic in environments with frequent topology changes and reducing the controller’s burden. In [22], the authors proposed a multi-path routing scheme, using multiple available paths in the network to forward traffic, balancing the network traffic on each path. In [23], the authors determined the deployment plan for controllers to achieve load balancing among controllers and a minimum total number of controllers by calculating the maximum tolerable delay for communication between switches and controllers, as well as among controllers. In [24], the authors proposed a load balancing and deployment plan for SDN network controllers by calculating the costs of monitoring module data collection, initial path request, flow table installation, controller information synchronization, and switch reallocation. In [25], Wang et al. continuously monitored the load of each controller in the network, dynamically changing the matching relationship between controllers and switches, that is, the reallocation of switches, to achieve network load balancing. In [26], the authors defined domain similarity rules to divide the entire SDN network into multiple highly similar domains, further deploying controllers at the logical centers of each domain to achieve the purpose of balancing network loads. In [27], Wang et al. modeled and processed the routing and redirection of flows to achieve maximum resource utilization and optimize network loads. In [28], Yang et al. used the idea of dynamically searching for the optimal path with ant colony algorithms to balance network loads. In [29], the authors proposed a load balancing algorithm based on symmetric hybrid routing to reduce link selection costs. To enhance the capability of the control plane and improve its robustness, the authors propose a distributed iterative strategy for multi-controller SDN traffic measurement, proven to converge to near-optimal performance, reducing switch load and controller communication [30].

With the rapid development of information technology, research in the field of networks has been continuously expanding and deepening, providing a rich background and reference for the study of path allocation in SDNs. In the category of communication technologies, visible light communication (VLC) and visible light laser communication (VLLC) have shown potential. For example, Reference [31] uses an integrated PD array device to achieve a 2 × 2 MIMO-VLLC link, improving the data rate. Reference [32] realizes ultra-high-speed short-range transmission through VLLC technology to meet the high-bandwidth requirements of data centers. In the area of the Internet of Things, the wireless multiferroic memristor proposed in Reference [33] integrates storage, processing, and communication, addressing the power consumption and latency issues of traditional IoT nodes. The network function virtualization (NFV) field has achieved fruitful results. Reference [34] proposes the SFCDO algorithm based on breadth-first search to optimize the end-to-end latency and resource consumption of SFC deployment. The heuristic closed-loop feedback CLF algorithm in Reference [35] improves the performance of network services while reducing costs. Reference [36] proposes effective methods to improve the success rate of SFC orchestration and save bandwidth for the SFC orchestration problem in multi-domain networks. In the field of vehicle-to-everything (V2X), Ref. [37] solves the interference problem in V2I communication by jointly designing the radar transmit waveform and receive filter bank.

Against this backdrop, the study of path allocation in SDN is of great significance for optimizing network resources and enhancing performance. It is expected to integrate the achievements of multiple fields and explore better path-allocation strategies.

Although numerous studies have been conducted in the field of path planning, there are still some unresolved issues. For example, in multi-end-to-end path request planning, the system may not be able to meet all path request requirements under limited bandwidth resources. Additionally, unreasonable path planning may lead to local network congestion and an imbalance in overall network load. Therefore, the multi-domain control plane needs to consider the status of more network resources during the path allocation process, including link weights, bandwidth overbooking factors, load saturation, resource occupancy rates, load skew, etc., in order to select the optimal paths to maximize the satisfaction of data plane requirements while maintaining network load balance. However, current research on cross-domain path selection under multi-end-to-end path requests does not sufficiently consider these factors.

To address such issues, we first derive a cross-domain communication load balancing objective function based on network modeling. Then, we transform the cross-domain path planning problem into a clique-finding problem under a set of backup paths. Finally, we provide a heuristic approximate solution method for this problem. In terms of cross-domain communication, we adopt a collaborative approach among multiple controllers to achieve coordinated planning of cross-domain paths and collaborative installation of flow tables.

## 2. System Model

This section models the path selection problem and defines relevant variables to provide a foundational theoretical model.

For any autonomous system (AS) in the data plane of a multi-domain SDN network, it can be equivalent to a connected graph $AS=\left(V,E\right)$, where $V=\left\{n_{1},n_{2},\cdots ,n_{N}\right\}$ represents the set of *N* physical nodes (switches or hosts) in the domain and $E=\left\{e_{ij}\right\}$ represents the set of communication links between all nodes, with $e_{ij}$ representing the link between nodes *i* and *j*. Here, *i* and *j* range from 1 to *N*, and the total number of links in the domain is $L<N^{2}$. The topology of domain *AS* is represented by the topology matrix *C* = [*C_ij_*], with *i* and *j* = 1, 2, …, *N*, where:

(1) $$C_{ij}=\left\{\begin{array}{c} 1,\;\;\;\;\;\;\;if\;n_{i}\;is\;connected\;to\;n_{j}\\ 0,\;\;\;\;\;\;\;\;\;\;\;\;otherwise\;\;\;\;\;\;\;\;\;\;\;\;\;\;\;\;\;\;\;\;\;\;\; \end{array}\right.$$

and by definition *C_ii_* = 1, ∀*i*. The Link Capacity Matrix is defined by *B* = [*B_ij_*] for the entire domain, where *B_ij_* represents the link capacity of link *e_ij_*. Clearly, the larger the value of *B_ij_*, the stronger the communication capacity of link *e_ij_*, and the more paths that can be allocated through *e_ij_*.

Assume the total number of end-to-end path requests received by the controller at the same time is *R*. For any path request denoted as (*S_r_*, *T_r_*, *b_r_*), where *r* = 1, 2, …, *R*, the corresponding path returned by the controller is represented by the vector $P_{r}=\left[V_{o}^{r},\;V_{1}^{r},\ldots ,V_{k_{r}}^{r}\right]$, which consists of *Kr* + 1 (*K_r_* > 1) directly connected nodes. These Kr + 1 nodes are connected from the source node $S_{r}=V_{0}^{r}$ to the destination node $T_{r}=V_{k_{r}}^{r}$. For any path *P_r_* at the *k*-th hop, with *k* = 1, 2, …, *K_r_*, it is from *V_k_*_−1_ to *V_k_*, and its bandwidth is denoted by *b_r_*.

For autonomous domain *AS*, its average path length is defined as [38]:

(2) $$L_{AS}=\frac{1}{N\left(N-1\right)}\sum_{i\neq j\in V}d_{ij}$$

And the domain’s internal cohesion degree is [38]:

(3) $$C_{AS}=\frac{1}{N\cdot L_{AS}}$$

where *N* = |*V*|(*N* > 1) represents the number of domain nodes; *d_ij_* represents the shortest path hop count between nodes *i* and *j*. *C_AS_* is used to reflect the cohesion degree of domain *AS* towards the logical center node. The larger its value, the more the domain AS is centralized overall, and conversely, the more dispersed the nodes within the domain. For ease of understanding, it can be intuitively considered that: *C_AS_* can be understood as the domain’s regional edge length, further understood as the domain’s regional size or area, and *N* refers to the number of nodes in that area. When *L_AS_* is constant and *N* increases, it indicates that the nodes within the domain are diffusing towards the edge, and the nodes within the domain are becoming more dispersed; when *N* decreases, it indicates that the nodes within the domain are contracting towards the center. When *N* is constant and *L_AS_* decreases, the nodes within the domain will compress towards the center, and the domain becomes more concentrated; when *L_AS_* increases, the domain stretches outward, and the domain becomes more dispersed. The domain’s internal cohesion degree reflects the degree to which the nodes within the domain are cohering towards the center.

**Figure 1:**
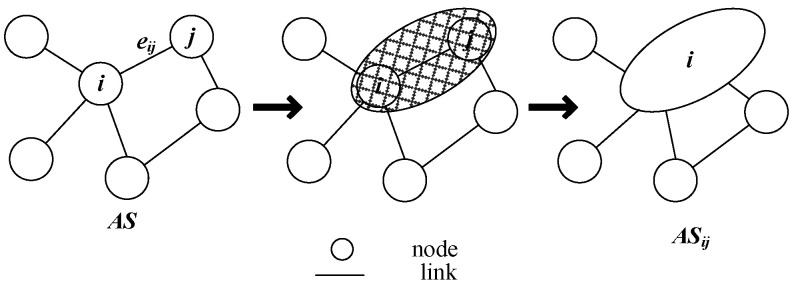
Domain *AS* migrates to *AS_ij_*.

At the same time, the migration graph *AS_ij_* based on edge *e_ij_* is defined, and its migration process is as follows: for any edge *e_ij_*, a new larger node is constructed to encompass *i*, *j*, *e_ij_* and replace their functions, meaning that all original connections related to *i* and *j* are replaced by the new node. At this time, *AS* will migrate to a new domain, denoted as *AS_ij_*. Figure 1 further illustrates the migration process based on edge *e_ij_*.

Furthermore, the weight factor *I_ij_* for each edge in the domain is defined as:

(4) $$I_{ij}=\frac{1}{A_{i}}(1-\frac{C_{AS}}{C_{AS_{ij}}})$$

where *A_i_* represents the average shortest path value from node *i* to all other nodes in domain *AS_ij_*. In Formula (4), the larger the value of *I_ij_*, the greater the weight of edge *e_ij_*. When the value of *I_ij_* is large enough, it is called a key edge. In the path selection process, the larger the value of *I_ij_*, the more paths edge *e_ij_* should carry.

For each responding path *Pr*, we define $X^{kr}=\left[x_{ij}^{kr}\right]$, where:

(5) $$x_{ij}^{kr}=\left\{\begin{array}{c} 1\;\;\;\;e_{ij}\in P_{r}\\ 0\;\;\;\;otherwise \end{array}\right.$$

and by definition $x_{ii}^{kr}=1$, ∀(*i*, *k*, *r*). When $x_{ij}^{kr}=1$, it means that the *k*-th hop in the allocated path *Pr* uses *e_ij_.*

For any edge *e_ij_* in domain *AS*, its load balance degree is represented by *D_ij_*, and the calculation method is:

(6) $$D_{e_{ij}}=e^{-\frac{\left|\xi B_{ij}I_{ij}-\sum_{r=1}^{R}\sum_{k=1}^{K_{r}}x_{ij}^{kr}\cdot b_{kr}\right|}{\xi B_{ij}I_{ij}}}$$

where ξ is the adjustment factor. Since $-\frac{\left|\xi B_{ij}I_{ij}-\sum_{r=1}^{R}\sum_{k=1}^{K_{r}}x_{ij}^{kr}\cdot b_{kr}\right|}{\xi B_{ij}I_{ij}}\leq 0$, it follows that $0\leq D_{e_{ij}}\leq 1$, ∀(*i*, *j*). The use of the power operation can more quickly enhance the load balancing degree of low-utilization links and more rapidly reduce the traffic of heavily loaded links.

It is worth noting that the link load optimization rate in this paper is optimal when it takes the value of 1. When the link load optimization rate is low, the link utilization may be low or high, at which point either the traffic on the link is too low or it has undertaken too much traffic.

In the multi-domain SDN network, the network load balancing and resource occupation optimization objective function is defined as:

(7) $$D^{G}=\sum_{\sigma =1}^{M}\zeta_{\sigma }\left(D_{\sigma }-\lambda H_{\sigma }\right)$$

where *M* represents the number of SDN domains, λ is the resource proportion weight coefficient. $\zeta_{\sigma }$, *D_σ_*, and *H_σ_*, respectively, represent the weight, domain load balance degree, and resource occupation value of the *σ*-th domain, and the calculation method of the domain load balance degree is as follows:

(8) $$D_{\sigma }=\frac{1}{2}\sum_{i=1}^{N}\sum_{j=1}^{N}D_{e_{ij}}^{\sigma }I_{ij}/w$$

where $D_{e_{ij}}^{\sigma }$ represents the load optimization rate of link *e_ij_* in the *σ*-th domain, $w=\sum_{i=1}^{N}\sum_{j=1}^{N}I_{ij}$ is used to normalize the variable $I_{ij}$, and is used to ensure that $D_{\sigma }\leq 1$.

The calculation method for resource occupation *H_σ_* is:

(9) $$H_{\sigma }=\sum_{r=1}^{R}\sum_{k=1}^{K_{r}}\sum_{i=1}^{N}\sum_{j=1}^{N}x_{ij}^{kr}$$

To facilitate processing, we assume that the weight of each domain is the same, i.e., let $\zeta_{\sigma }=\frac{1}{M},\;\forall \sigma$, thus simplifying Formula (7) to:

(10) $$D^{G}=\frac{1}{2M}\sum_{\sigma =1}^{M}\sum_{i=1}^{N}\sum_{j=1}^{N}\left(D_{e_{ij}}^{\sigma }I_{ij}/w-\lambda \sum_{r=1}^{R}\sum_{k=1}^{K_{r}}x_{ij}^{kr}\right)$$

Obviously, in order to maximize Formula (7), it is necessary for $D_{\sigma }$ in each domain to be as large as possible and $H_{\sigma }$ to be as small as possible. That is, the more balanced the load in each domain, the fewer network resources (path hops) used, and the larger the value of Formula (7). Conversely, the larger the value of Formula (7), the more balanced the network load, and the fewer path hops are occupied.

Since $D_{\sigma }\leq 1$, $H_{\sigma }$ is an integer and the minimum increment is 1, therefore when *λ* ≥ 1, maximizing Formula (7) is equivalent to prioritizing the selection of paths with the shortest number of hops (i.e., paths with small $H_{\sigma }$ values, and $H_{\sigma }$ decreases by at least 1 each time) in path planning, and then selecting the most balanced network load path (paths with small $D_{\sigma }$ values) from multiple shortest paths. When 0 < *λ* < 1, the increment step length of $\lambda H_{\sigma }$ may not be greater than 1, so (7) does not prioritize the selection of the shortest hop count or optimal load in path planning but comprehensively considers the weighted value $D_{\sigma }-\lambda H_{\sigma }$. When *λ* = 0, the path selection process will ignore the resource occupation situation and only consider the network load balance degree. It can be seen that *λ* plays a role in dynamically adjusting the proportion of resource occupation and network load.

## 3. Cross-Domain Communication Method Based on Load Balancing for SDN Networks

**Figure 2:**
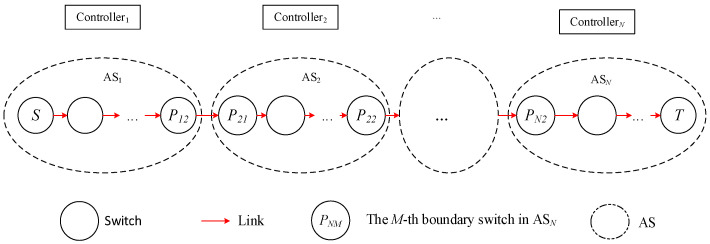
Communication path of cross-domain.

In actual SDN networks, data are often transmitted across domains, so a complete path from the source node to the destination node will pass through multiple domains. The domain control restriction in SDN means that each domain controller can only plan the paths within its domain, and these paths are often just a part of the complete path, as shown in Figure 2 below.

In Figure 2, the complete path (*S*, *T*) goes through *N* domains, with the source node being node S in domain 1 and the destination node being node *T* in domain *N*.

**Figure 3:**
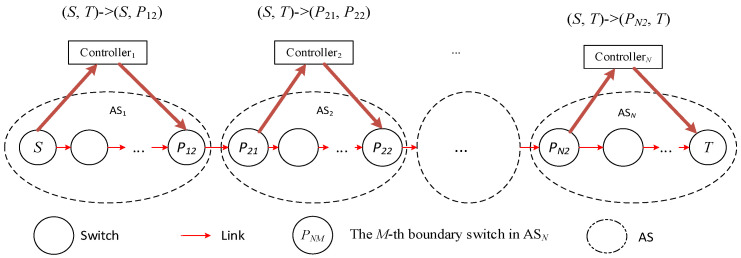
Decomposition of cross-domain path.

When the source node *S* needs to transmit data to the destination node *T*, it sends a path allocation request to controller 1. After receiving the path request, controller 1 first determines the domain *N* to which node *T* belongs based on global information and then learns that data must pass through domain 2 to reach node *T*. At this point, the controller identifies the boundary switch *P*_12_ between domain 1 and domain 2, then converts the original request path (*S*, *T*) to (*S*, *P*_12_), and plans the path $S\to P_{12}$ and installs the flow table, as shown in Figure 3. Data arrives at node *P*_12_ from *S*, then reaches *P*_21_, and at this point, *P*_21_ continues to request path allocation from controller 2, and so on, as shown in Figure 3.

**Figure 4:**
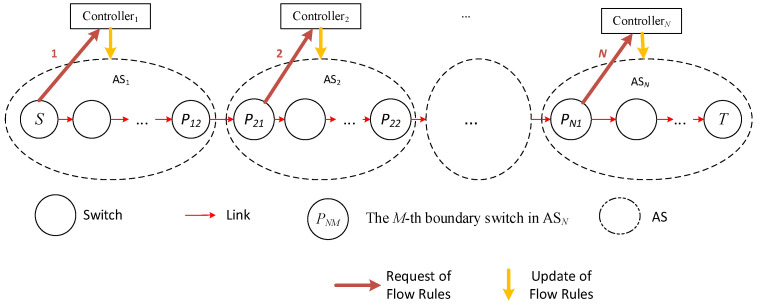
SDN network cross-domain communication.

In this process, *N* path requests, path planning, and flow table issuance operations occur; initiated by switches *S*, *P*_21_, …, *P_N_*_1_ (as shown in Figure 4), which is the mainstream method of flow table request and issuance in a multi-domain environment.

**Figure 5:**
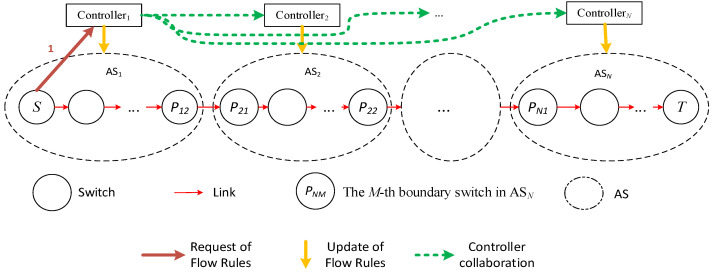
Path decomposition and planning for multi-controller collaboration.

To balance the network load and reduce network communication latency, this paper will improve the above communication process. Taking the same path (*S*, *T*) request and domain scenario as shown in Figure 4 as an example, when the source node *S* needs to transmit data to the destination node *T*, it first sends a communication request to controller 1. After receiving the flow table request, controller 1 plans the path and issues the flow table. At the same time, controller 1 directly sends data to controllers 2~*N* via high-speed links (assuming controllers are connected by high-speed links) to inform them that data for (*S*, *T*) will pass through, and then controllers 2~*N* collaborate on path decomposition and planning to allocate the optimal path scheme, i.e., maximizing Formula (7), and pre-issue flow tables to relevant switches within the domain as shown in Figure 5.

In the above process, only one communication request operation occurs; that is, S sends a request to controller 1, and the other *N* − 1 communication request operations are completed by direct communication between controller 1 and controllers 2~*N*. It can be seen that when all switches on the path *S* to *T* lack flow table entries, compared with the traditional method shown in Figure 2, the multi-domain SDN network communication method proposed in this paper reduces the number of network communication request operations, and network load balancing can be achieved through controller collaboration, which helps to reduce the end-to-end latency of network cross-domain communication and the average latency of overall network communication.

**Figure 6:**
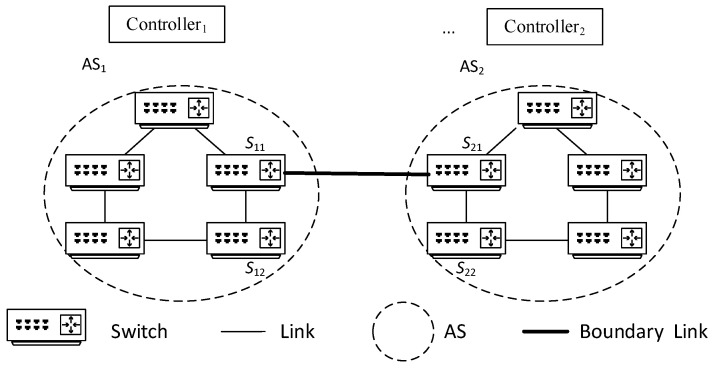
Network topology with a unique boundary forwarding node.

In cross-domain path requests, due to different network topologies, the selection of boundary forwarding nodes will present various situations. For this issue, inter-domain routing can be discussed in three cases:

(1) The boundary forwarding node is unique, as shown in Figure 6

In this case, all cross-domain communication packets will be directly forwarded to the unique boundary forwarding node, and then the packets will be forwarded to the next autonomous domain by that node. This is a situation where inter-domain routing selection is not required.

**Figure 7:**
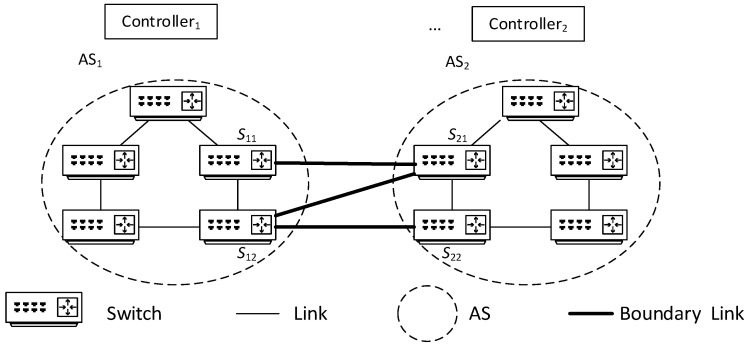
The adjacent domains have multiple boundary forwarding nodes.

(2) The boundary forwarding links are not unique, and the next hop points to the same autonomous domain (as shown in Figure 7)

In this case, regardless of the choice of boundary nodes, packets will be forwarded to the next autonomous domain. Macroscopically, the choice of boundary nodes will not affect the total load of the next autonomous domain and cannot achieve inter-domain load balancing. Microscopically, the choice of boundary nodes and their transmission ports determines the data transmission path of packets in the current domain and the next domain, which can affect the balance within the domain.

For this issue, this paper defines a calculation formula *f*(*h*, *p*, *b*, *r*) for the selection of boundary forwarding nodes and transmission ports for inter-domain routing decisions. Where *h* is the shortest path hop count from the path request node within the domain to the boundary forwarding node, *p* is the number of packet transmissions at the boundary forwarding node, *b* is the byte forwarding count at the boundary forwarding node, and *r* is the port data transmission rate of the boundary forwarding node’s inter-domain communication link. The implementation of this formula is a weighted sum of *h*, *p*, *b*, and *r*. The weights are determined by the network administrators of each domain; the larger the value of *f*, the better the link. Since the internal network topologies of different autonomous domains are different, and the application scenarios and requirements are different, it is impossible or very difficult to find a universal boundary calculation selection method that applies to different autonomous domains. In actual applications, *f* can be determined through configuration files and inter-domain controller collaboration to assist in the selection of boundary forwarding links.

(3) The boundary forwarding links are not unique, and the next hop points to multiple different autonomous domains

**Figure 8:**
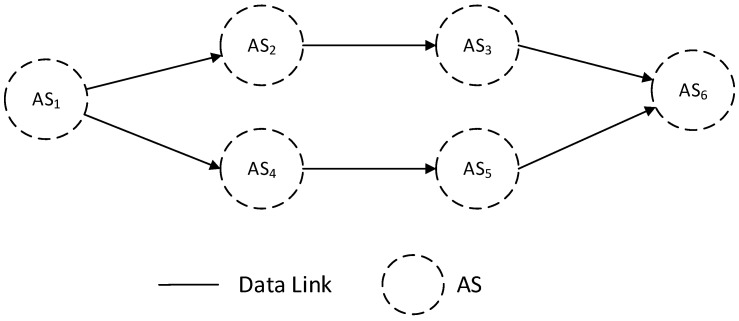
Boundary forwarding nodes are not unique and point to different domains.

In this topology, the inter-domain routing decision problem can be transformed into a next-domain selection problem. By abstracting each autonomous system as a node, the problem is further converted into a path selection problem based on domain nodes. For example, in Figure 8, when data reaches the boundary of Domain 1, it can choose the next hop to be either Domain 2 or Domain 4. If it is Domain 2, then the data transmission path is 1 -> 2 -> 3 -> 6. If it is Domain 4, the path is 1 -> 4 -> 5 -> 6. During network operation, since the load status of each domain is dynamically changing, the selection of the next domain for data forwarding should also be dynamically determined (for example, if domain 2 is congested, domain 4 should be chosen as the next domain). To address this issue, we set a round-trip delay threshold range $\left[{\underline{\lambda }}_{m,n},{\bar{\lambda }}_{m,n}\right]$ (${\underline{\lambda }}_{m,n}<{\bar{\lambda }}_{m,n}$) for the selection of the next domain and introduced a domain priority parameter $k_{m,n}$ to dynamically assist in determining the selection of the next domain. Here, $k_{m,n}$ represents the priority of domain *n* relative to domain *m*, that is, the priority value of domain *n* when domain *m* selects the next domain. ${\underline{\lambda }}_{m,n}$ and ${\bar{\lambda }}_{m,n}$ are the minimum and maximum delay thresholds from domain *m* to domain *n*, respectively. For ease of explanation, we define $t_{m,n,d}^{s}$ to represent the communication delay of packet *s* on the path from domain *m* to domain *n* and so on to domain *d*. Then, $t_{m,n,d}^{s}+t_{d,n,m}^{s}$ is used to denote the round-trip delay of the packet. During network operation, each domain controller readjusts the domain priority parameters at regular intervals or makes on-demand adjustments to the domain priority parameters when congestion is detected. The adjustment method is:

(11) $$k_{m,n}=k_{m,n}-\alpha (e^{t_{m,n,d}^{s}+t_{d,n,m}^{s}-{\bar{\lambda }}_{m,n}}-e^{{\underline{\lambda }}_{m,n}-t_{m,n,d}^{s}-t_{d,n,m}^{s}})$$

where *α* is the adjustment factor, and *s* is the packet sent from domain *n* to domain *d*. It can be seen that the higher the round-trip delay of packet *s*, the faster the priority value of domain *n* decreases, and vice versa, the faster it increases. Combining the domain priority parameter *k* and the *h*, *p*, *b*, and *r* mentioned above, a new calculation formula is redefined for selecting the boundary forwarding link, where *k* is the priority value of the next domain corresponding to the link, and *f* can be most directly implemented as the weighted sum.

Taking Figure 8 as an example, if $\left(t_{1,2,6}^{s}+t_{6,2,1}^{s}\right)>{\bar{\lambda }}_{1,6}$ indicates that the path 2 -> 3 -> 6 is congested, then Formula (11) will reduce the priority value $k_{1,2}$ of Domain 2 relative to Domain 1, and the larger the value of $t_{1,2,6}^{s}+t_{6,2,1}^{s}$, the faster the $k_{1,2}$ value will decrease. In this way, we can indirectly reduce the level of network congestion by migrating the load between domains.

## 4. Problem Analysis and Algorithm

Assuming *R* is the number of end-to-end path requests received by domain AS*_i_* (*i* = 1, 2, …, *M*) at the same time. After inter-domain decomposition, for any end-to-end path request (*S_r_*, *T_r_*), where 0 < *r* ≤ *R*, *r* = 1, 2, …, *R*, it is assumed that the controller of AS*_i_* stores *p_r_* backup optional paths for communication between (*Sr*, *Tr*). For all *R* path requests in domain AS*_i_*, the set of backup paths is defined as $AltPath^{R}=\{P_{n}^{r}\;|\;r\;=1,\;2,\cdots ,\;R\}$, for a given *r*, let *n* = 1, 2, …, *p_r_*, where $P_{n}^{r}$ represents the *n*-th (*n* < *p_r_*) backup path for path (*S_r_*, *T_r_*), and the total number of *R* backup paths is $\left|AltPath^{R}\right|=\sum_{r=1}^{R}p_{r}$.

Combining the system model, it can be known that the optimal path planning scheme is equivalent to each domain finding the optimal *subset*({$P_{n}^{r}$}) from the set of backup paths ({$P_{n}^{r}$}) for the communication of *R* end-to-end path requests, and this set can maximize the value of Formula (7).

**Figure 9:**
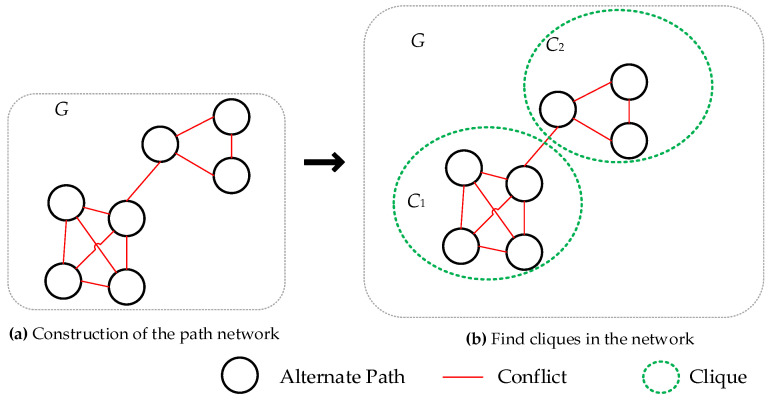
Clique problem to our path assignment.

Since there are interdependent relationships between each backup path due to the resources they occupy, the method of constructing a new network graph by taking backup optional paths as nodes and the exclusive relationships between backup paths as edges approximates the path selection problem to the problem of finding all cliques in graph theory (the clique problem has strong inapproximability characteristics). We first construct a new path network *G* in the manner of Figure 9a. For any backup optional path *p* corresponding to a node in the generated network, if there exists another backup path $q\in \left\{P_{n}^{r}\right\}$ and *p* conflicts with *q* in path allocation, then an edge *e_pq_* is created in network *G*.

After the network is constructed, for any clique *c* in network *G* as shown in Figure 9b, including cliques *c*_1_, *c*_2_, select a node *q* ∈ *c*, and *q* is the node in clique *c* that can maximize the value of Formula (7) and is not directly connected to the neighboring cliques. Proceed in this manner, selecting one node from all cliques to form a path allocation set.

In the above narrative, by constructing a new network graph with backup optional paths as nodes and exclusive relationships between backup paths as edges, the multi-objective optimization path selection problem is approximated to the problem of finding all cliques in graph theory, and the optimal path allocation set is formed by finding the best and non-conflicting paths in each clique. Below, an approximate algorithm for the path planning problem will be given (Algorithm 1).
**Algorithm 1.** Cross-domain Communication Path Assignment (CCPA)Let $S=P_{n}^{r}$;//Each domain controller defines a set of optional backup paths within the domain, denoted as $S=P_{n}^{r}$. This set is precomputed by each controller and stored in memory.Let *R* =0;Let L=∅;RepeatFetch the *r*-th path request *P_r_*;Perform domain decomposition on *P_r_* to generate a new multi-domain path request $P_{r}^{\prime }$;Select an path *p* from *S* for the path request $P_{r}^{\prime }$ such that *p* is not in *L* and can maximizes Equation (7);Add *p* to *L*;Remove *p* from *S*;For all ∀q∈S, if *q* conflicts with any path in *L*, remove *q* from *S*;*r*++;Until |*L*| >= *R* || S==∅Output *L*;

In the process of path request and selection within each autonomous domain *AS*, for any path request (*S*, *T*) within the domain, it is assumed that *N* paths can be found in *AS*, However, a large portion of these *N* paths are discarded due to excessive hop counts. Define the path’s overlength factor *h*, assuming the shortest hop count between nodes *S* and *T* is *H_ST_*. In actual path selection, if the path length from *S* to *T* exceeds *H_ST_* + *h*, then discard that path. The AS controller can pre-store the backup paths from each source node to the destination node. Obviously, if the total number of backup paths is assumed to be *L*, *L* must be much smaller than *N*. As mentioned above, the path allocation process is actually the process of selecting the optimal path from the set of backup paths. In extreme cases, if there is only one backup path from all source nodes to destination nodes, then without considering the bandwidth, the path allocation will be fixed. And if all backup paths are the shortest paths, then the path allocation result will be consistent with that of the traditional network (paths generated by the Dijkstra algorithm). Since *L* is much smaller than *N*, the computational complexity of the algorithm will not be large, and thus the operational efficiency of the path allocation algorithm will not be poor.

## 5. Performance Evaluation

### 5.1. Simulation Assumptions

To verify the feasibility of the load balance-oriented cross-domain communication method proposed in this paper, the simulation will be conducted in a multi-domain environment. The performance indicators of the network include: (1) Path allocation success rate; (2) Network load balance degree; (3) Average delay of cross-domain communication. The path allocation success rate indicates the proportion of successful returns of valid paths by the controller after receiving the path requests. It is worth noting that the bandwidth requested for the allocated path must not exceed the remaining bandwidth of the link; otherwise, the path allocation will not be successful. The network load balance degree represents the load balance of the entire network after the paths are allocated. The average delay of cross-domain communication represents the average delay of all cross-domain data packets from the source node to the destination node.

In the simulation, the number of nodes in each domain that can send and receive data and the links between nodes are randomly generated. During the topology generation process, for any node, other nodes are randomly selected to connect.

The bandwidth of each link in the network is randomly generated within the range [5 × 10^4^, 10^5^]. Each end-to-end path request (*S*, *T*) is randomly selected from the network nodes. Additionally, for any path request (*S*, *T*), its bandwidth request is randomly generated within the range [1, 5 × 10^4^ × *Linkfactor*], where *Linkfactor* is the network bandwidth request pressure intensity factor, and the value range is set to [0, 1]. The larger the *Linkfactor* is set, the higher the network bandwidth demand is. Conversely, it will have a lower network bandwidth demand.

Furthermore, for any link, the remaining bandwidth of the link must be greater than the bandwidth requested by the path. This means that when the controller allocates a path, it must consider the bandwidth constraints of each link. The bandwidth requirement of the allocated path should be less than the remaining bandwidth of all the links included in that path.

**Figure 10:**
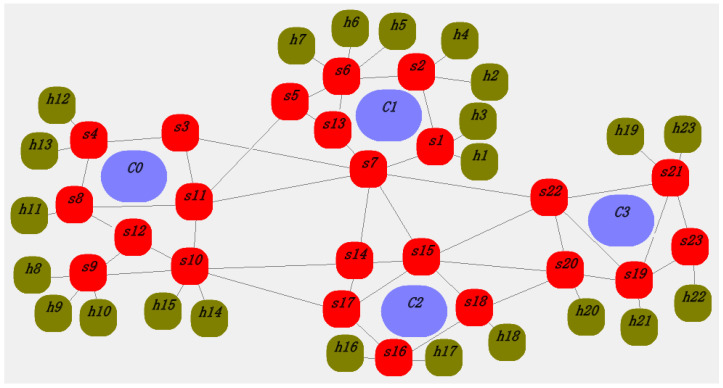
Network topology.

The total number of path requests per second in each domain is *N* × (*N* − 1) × *NetworkStress*, where *NetworkStress* is the path request pressure factor. Each controller will receive *N* × (*N* − 1) × *NetworkStress* randomly generated path requests. The larger the *NetworkStress* is set, the higher the overall network path request pressure intensity is. The network runs 200 times to generate average data for each performance indicator, and the topology is shown in Figure 10. The specific values of each parameter are shown in Table 1.

In the simulation, the shortest path communication method under the traditional cross-domain communication mode as shown in Figure 4 (referred to as CD-SPF) and the load balance-oriented cross-domain communication method proposed in this paper as shown in Figure 5 are compared.

In Figure 10, *s*1 to *s*23 are OpenFlow switches, *h*1 to *h*23 are terminal host nodes, and *c*0 to *c*3 are domain controllers. Taking domain 0 as an example, it consists of controller *c*0, switches *s*3, *s*4, s8, *s*9, *s*10, *s*11, *s*12, and terminal nodes *h*8 to *h*15. Other domains are similar and will not be described in detail here.

**Figure 11:**
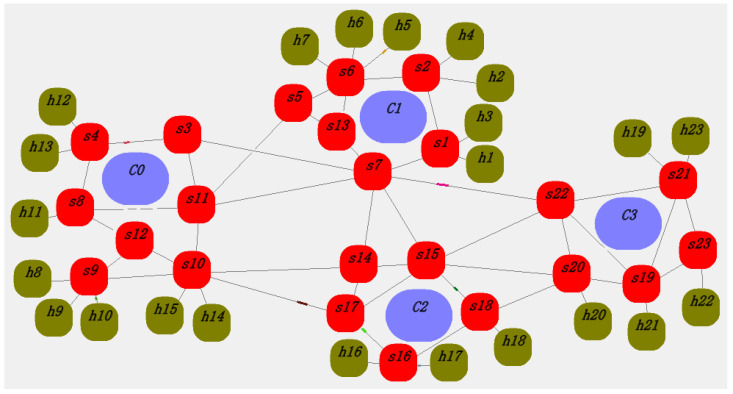
Network communication screenshot 1.

**Figure 12:**
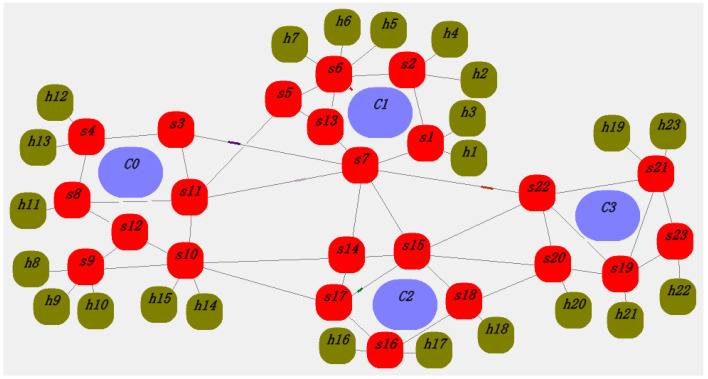
Network communication screenshot 2.

The data transmission rate for each switch, terminal node, and controller is set to 100 M. The packet length is a random value between 64 and 1518 bytes. The length of the flow table request packet is set to 64 B, and the length of the flow table installation packet is set to 64–128 B. The flow table is distributed using direct communication between the controller and the switch. Figure 11 and Figure 12 are random screenshots during the simulation process. The thick red lines between the controller and the switch (such as s6, c1 in Figure 12) indicate that the switch is requesting flow tables from the controller (packet-in events) or the controller is updating flow rules to the switch (packet-out events).

### 5.2. Simulation Results and Analysis

To more accurately and intuitively observe the results of path allocation, in the simulation, we assume that during the path allocation process, the requested bandwidth of the allocated path cannot exceed the remaining bandwidth of each link traversed by the path; otherwise, the path allocation is deemed unsuccessful. Additionally, to ensure that domain controllers are actively involved in the communication process, we assume that all switch flow rules become invalid after being used once, meaning that all packets generate a packet-in event. In contrast, traditional network experiments do not have these constraints. It is widely recognized that the shortest path first (SPF) algorithm can identify the shortest communication path in a network. Given its simplicity, efficiency, and intuitiveness, it has consistently served as the benchmark algorithm and a common strategy for path planning in network communications. For multi-domain SDN networks, when factors such as negative domain balancing, multi-path support, and network throughput are not considered, cross-domain shortest path first (CD-SPF) can be regarded as the preferred strategy for path allocation. Due to the different simulation assumptions compared to existing research methods, the simulation only compares with the CD-SPF algorithm to verify its performance metrics in terms of path allocation success rate, network load balancing, end-to-end average latency, and flow table installation time.

During network operation, the performance metrics of different schemes are tested by varying the network bandwidth request pressure and the path request pressure factor.

**Figure 13:**
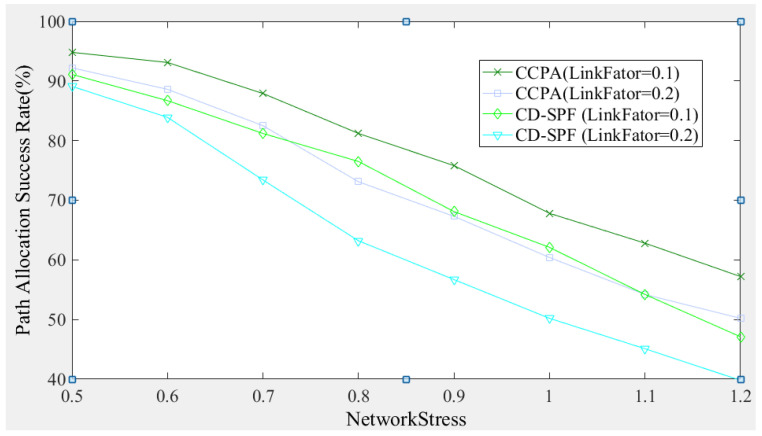
Comparison of path allocation success rate.

Figure 13 compares the performance of the proposed method (CCPA) and the CD-SPF algorithm in terms of path allocation success rate. The path allocation success rate fluctuates within the range of [39.7~94.8%]. As the number of path requests increases, the proposed algorithm generally has a higher path allocation success rate than CD-SPF. When *LinkFactor* = 0.1, the difference between the two methods is approximately 5% to 10%, and this gap tends to widen as *NetworkStress* increases. When *LinkFactor* = 0.2, the allocation success rate of CCPA is about 11% higher than the best performance of CD-SPF. The overall trend shows that under different *LinkFactor* values, as the number of path requests increases, the path allocation success rate decreases for both methods. When the number of path requests exceeds a certain value and network resources (bandwidth and number of available links) are limited, the available resources for path allocation decrease with the increase in *LinkFactor* and *NetworkStress*, leading to a lower path allocation success rate, which tends to decrease linearly. This phenomenon indicates that increasing the available network resources or reducing *LinkFactor* and *NetworkStress* can improve the path allocation success rate.

**Figure 14:**
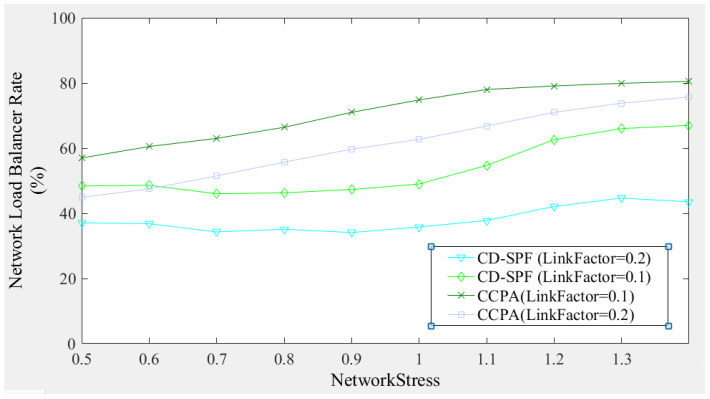
Comparison of network load balancing rate.

Figure 14 compares the proposed method and CD-SPF in terms of network load balancing performance. When *LinkFactor* = 0.1 and *NetworkStress* = 1.0, CCPA achieves a load balancing degree close to 75%, while CD-SPF is around 48%. When *LinkFactor* = 0.2, the network bandwidth request is relatively larger, and as *NetworkStress* increases, CD-SPF’s network load balancing degree does not improve significantly, whereas CCPA can maintain a stable increase in network load balancing. The figure shows that under different network bandwidths and path requests, the proposed method achieves a more balanced network load, indicating that CCPA improves the utilization of network bandwidth resources in path allocation.

**Figure 15:**
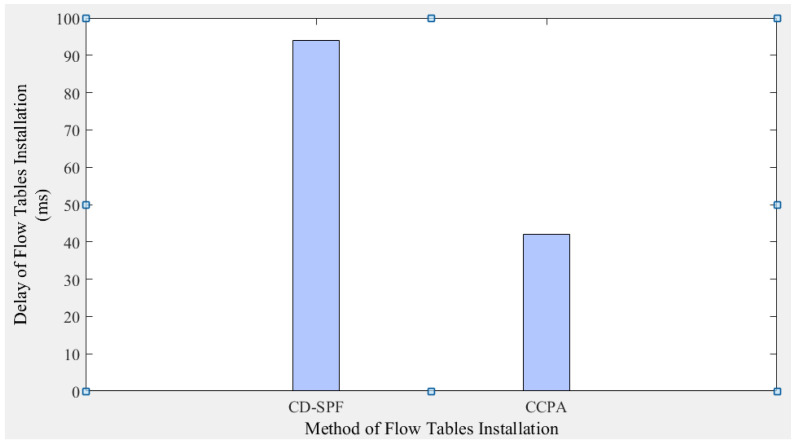
Comparison of flow tables installation delay.

Cross-domain path data transmission mainly includes three stages: (1) path request; (2) path selection and flow table installation; (3) packet forwarding. The path selection results are generated by internal calculations of each domain controller, and the calculation time is strongly related to the hardware performance of the controller. In the statistics of end-to-end delay, this simulation ignores computational delay; that is, it disregards the computational delay of the controller in path planning. In terms of packet-in and packet-out events, the flow table request and installation time for CD-SPF (Figure 4) and the proposed method (Figure 5) are shown in Figure 15. CD-SPF has an average latency of about 94 ms in cross-domain communication, while the proposed method is around 42 ms.

**Figure 16:**
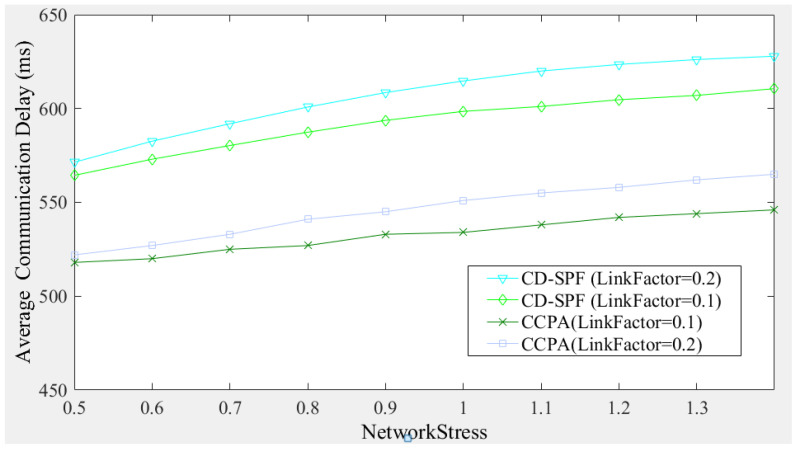
Comparison of average cross-domain communication delay.

**Figure 17:**
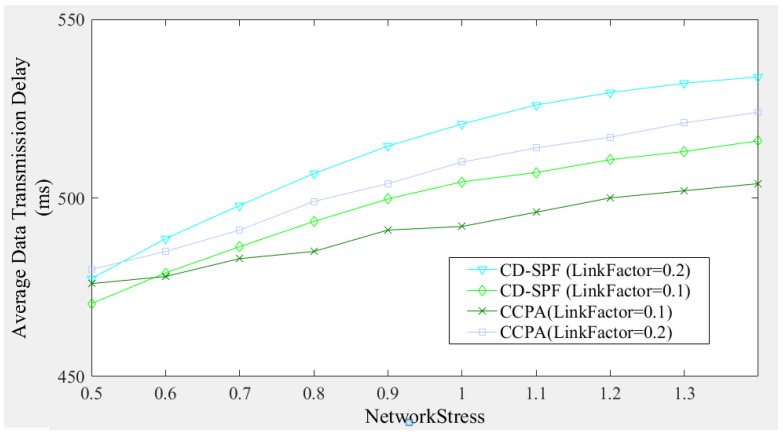
Comparison of average cross-domain data transmission delay.

Since the simulation assumes that switch flow rules become invalid after being used once (to simulate a high-density path request environment), all cross-domain communication packet-in events will trigger the domain controller to install flow rules for the switches in the path. Therefore, the cross-domain communication latency will include both flow rules installation latency and cross-domain data transmission latency. Figure 16 compares the proposed method and CD-SPF in terms of cross-domain communication latency. To further observe the impact of paths and loads on cross-domain communication, the data difference between Figure 15 and Figure 16 was used to derive the comparison of cross-domain data transmission delay shown in Figure 17. In the cross-domain data transmission latency comparison chart, it can be seen that when the network pressure is relatively low (for example, *NetworkStress* < 0.8), network resources are relatively abundant. At this time, CD-SPF has some advantages in terms of transmission delay due to the shortest number of path forwarding hops. However, as the number of path requests increases and available network resources become limited, when *NetworkStress* > 1.1, the proposed method has a lower average cross-domain data transmission latency, and when *LinkFactor* = 0.2, the proposed method’s performance in latency can surpass CD-SPF more quickly. According to the data results, when network bandwidth is sufficient, CD-SPF can leverage its shortest path advantage, but when the network experiences a certain degree of congestion, CD-SPF’s advantage gradually diminishes. In contrast, the proposed load balancing method begins to show its advantages. This is because the proposed method can balance the network load across all links, thereby reducing the overall average transmission delay.

In summary, our proposed method outperforms the traditional method in terms of path allocation success rate, network load balancing degree, and data transmission delay, especially in cross-domain communication under high-density path requests in SDNs networks.

## 6. Conclusions

This paper focuses on the cross-domain communication problem based on load balancing in multi-domain SDN networks. Through network modeling, the problem of cross-domain load balancing planning for paths is transformed into a clique-finding problem for backup paths, and an approximate algorithm is provided. Through collaborative processing among multi-domain controllers, the coordinated planning of cross-domain paths and the collaborative installation of flow tables are achieved. Simulation comparisons show that the method proposed in this paper has a significant advantage in cross-domain communication under high-density path requests in SDN networks. In this paper, to more accurately measure the end-to-end delay, we have neglected the computational delay of the controller, which will be addressed in our future work. In future research, we plan to explore the integration of machine learning techniques into cross-domain path selection to enhance the success rate of path allocation and the load balancing rate across domains. Furthermore, we intend to investigate network path allocation strategies tailored to specific industrial fields, enabling the protocol to effectively meet the communication needs of different industrial sectors and better serve the relevant industries, especially the cyber-physical systems with high real-time communication requirements.

## Figures and Tables

**Table 1:** Simulation parameters.

Parameter	Values
Link Bandwidth	5 × 10^4^~10^5^
Path Request Bandwidth	1~5 × 10^4^
*NetworkStress*	0.5~1.3
*LinkFactor*	0.1, 0.2
Path Overlength Factor *h*	4
Link Bandwidth Surplus Factor	1
λ	1

## Data Availability

Data are contained within the article.
